# Depressive symptoms among older adults in Turkey: Evidence from a nationally representative ageing survey

**DOI:** 10.1371/journal.pone.0354722

**Published:** 2026-07-30

**Authors:** Zeynep Sedef Varol

**Affiliations:** Department of Public Health, Izmir Democracy University Faculty of Medicine, Izmir, Turkey; Taipei Medical University, TAIWAN

## Abstract

**Background:**

Late-life depression is a growing public health issue in ageing societies, influenced by health, functional, socioeconomic, and psychosocial factors. Evidence integrating health, functional, psychosocial, and healthcare-access determinants using nationally representative data in Turkey remains limited. This study examined determinants of depressive symptoms among adults aged ≥65 years.

**Methods:**

This study analyzed data from 10,348 participants aged ≥65 years from the nationally representative Turkey Older Persons Profile Survey (TYPA 2023). All analyses incorporated survey sampling weights. Depressive symptoms were defined using the Geriatric Depression Scale-30 (GDS-30) (cut-off ≥10). Hierarchical logistic regression models were constructed in sequential blocks (sociodemographic; health and functional status; psychosocial and healthcare-related factors) to identify independent correlates.

**Results:**

The weighted mean age was 72.8 years; 55.1% were women, and 46.2% screened positive for depressive symptoms according to the applied screening threshold. In the fully adjusted model, depressive symptoms were associated with female sex, lower education, unmarried status, poorer self-rated health, sensory and functional limitations, perceived age-related restriction, perceived exclusion, and difficulties accessing or communicating with healthcare services.

**Conclusions:**

Depressive symptoms among older adults in Turkey reflect the combined influence of health decline, functional limitations, negative psychosocial perceptions of ageing, and barriers to healthcare access. Interventions addressing functional decline, financial strain, ageism, and access barriers may support better mental health in later life and inform public health and ageing-related policies.

## Introduction

The rapid global growth of the older population has increased the prevalence of chronic diseases, functional decline, and social isolation, making mental health problems—particularly depressive symptoms—a major public health concern among older adults [1]. Depression in later life is associated with reduced quality of life, impaired daily functioning, increased healthcare utilization, and higher mortality [2]. Despite this burden, depressive symptoms in older adults often remain unrecognized because they are masked by somatic complaints, cognitive changes, or the tendency to normalize emotional distress as part of ageing [3].

Validated screening tools are therefore essential for early identification of depressive symptoms in older populations. The Geriatric Depression Scale (GDS-30), developed by Yesavage et al. [4], is widely used internationally because it is easy to administer, minimally influenced by somatic symptoms, and suitable for use in individuals with multimorbidity. The scale has been validated across multiple cultural settings, and studies consistently report strong psychometric properties and good screening performance with cut-off scores between 9 and 11 [5,6]. Turkish validation studies have also demonstrated the reliability of the GDS-30 in older adults [7]. Beyond its psychometric strengths, the GDS-30 is particularly suitable for population-based ageing research, as it facilitates large-scale screening in community-dwelling older adults.

Depressive symptoms in older adults are shaped by a wide range of sociodemographic, health, economic, behavioral, and psychosocial factors. Previous research has shown that advanced age, female sex, low education, multimorbidity, functional limitations, financial strain, social isolation, and ageism increase vulnerability to depression in later life [8–10]. However, nationally representative studies integrating these multiple domains within a single analytic framework remain limited in Turkey.

Turkey Older Persons Profile Survey (TYPA 2023), conducted by the Turkish Statistical Institute, provides comprehensive nationally representative data on the health, social environment, and living conditions of adults aged 50 years and older [11]. This dataset offers a unique opportunity to examine depressive symptoms and their determinants using standardized measures in a large, population-based sample.

Countries undergoing rapid demographic ageing and healthcare system transformation may exhibit distinct patterns of vulnerability in later-life mental health. Turkey represents an important case in this context. Over the past two decades, the country has experienced rapid population ageing alongside major reforms in healthcare delivery and social policy, including the Health Transformation Program that substantially expanded healthcare coverage and service availability. While improvements in healthcare infrastructure have increased service availability, older adults may still encounter barriers related to communication with healthcare providers, social participation, and perceived age-related exclusion [12]. Examining depressive symptoms within this structural and social context may therefore provide important insights into the determinants of late-life mental health in rapidly ageing societies.

Therefore, this study aimed to assess depressive symptoms among adults aged 65 years and older in Turkey using the GDS-30, and to identify sociodemographic, health-related, economic, and psychosocial factors associated with depressive symptoms. Using a hierarchical modelling approach applied to nationally representative survey data, this study contributes to filling an important gap in the Turkish geriatric mental health literature.

## Materials and methods

### Study design and data source

This study utilized anonymized microdata from the Turkey Older Persons Profile Survey (TYPA 2023), a nationally representative cross-sectional survey conducted by the Turkish Statistical Institute in collaboration with the Ministry of Family and Social Services [11]. TYPA 2023 was the first nationally representative older persons profile survey conducted in Turkey. Fieldwork was carried out between 23 October and 18 December 2023. A total of 22,640 households containing at least one individual aged 50 years or older were selected, and information was collected from 29,785 individuals aged ≥50 years, including 11,657 adults aged 65 years and older. The survey was designed using a stratified multistage probability sampling framework covering all 12 NUTS-1 (Nomenclature of Territorial Units for Statistics Level 1) regions, using a two-stage cluster sampling design. Data collection was conducted by TurkStat’s 26 Regional Directorates using computer-assisted personal interviewing (CAPI) and, when necessary, computer-assisted telephone interviewing (CATI). The survey aims to provide nationally representative information on the health status, living conditions, and social characteristics of older adults in Turkey.

Individuals unable to complete the interview because of cognitive, physical, or communication limitations could be represented by proxy respondents in TYPA 2023. However, subjective mental health measures, including the GDS-30, were administered only to participants who completed the interview themselves. Consequently, proxy-completed interviews were excluded from the present analysis because depressive symptoms could not be validly assessed for these individuals. After excluding proxy-completed interviews and records with missing GDS-30 data, the final analytic sample consisted of 10,348 older adults.

### Theory-informed analytical framework

The selection and organization of variables were guided by established gerontological and mental-health models. These models conceptualize late-life depression as the outcome of interacting structural, health-related, economic and psychosocial factors.

Sociodemographic and life-course factors (age, sex, education, marital status) represent structural determinants that shape vulnerability to depressive symptoms across the life course. Longitudinal analyses demonstrate that socioeconomic trajectories and marital history have enduring effects on depressive symptom patterns in later life [13].

Second, health status and functional impairments reflect the “disablement process”. Within this framework, chronic diseases and age-related physiological decline lead to mobility, sensory, cognitive and daily activity limitations that increase depression risk. Evidence consistently shows strong associations between self-rated health, functional loss and late-life depression [14].

Third, economic strain and healthcare access barriers represent contextual environmental stressors that restrict autonomy and reduce coping capacity. International reviews show that financial hardship, material deprivation and difficulty accessing healthcare services are major determinants of depressive symptoms among older adults [15,16].

Finally, psychosocial perceptions of ageing—such as feeling limited by age or perceiving age-based exclusion—are increasingly recognized as independent predictors of depression and poorer psychological well-being in later life. Negative age-related attitudes and perceived ageism have been systematically linked with depressive symptoms [17].

Based on this framework, a hierarchical modeling strategy was implemented. Variables were entered in theoretically informed blocks. Model 1 included structural sociodemographic determinants reflecting life-course social position. Model 2 added health and functional limitations capturing the disablement process. Model 3 incorporated economic stressors, healthcare barriers and psychosocial perceptions of ageing, representing contextual and subjective determinants of late-life mental health.

Based on this conceptual framework, all study variables were selected a priori and assigned to model blocks according to their theoretical relevance. Sociodemographic variables were included as structural determinants, health and functional indicators represented the disablement process, and economic, healthcare-access, and psychosocial variables reflected contextual and subjective influences on mental health. Variable selection was therefore guided by conceptual considerations rather than solely by statistical significance.

This layered approach acknowledges that late-life depression is associated with multiple interacting influences. These include long-term social positioning, declining intrinsic capacity, environmental constraints, and subjective perceptions of ageing.

### Measures

Dependent Variable: Depressive symptoms were assessed using the 30-item Geriatric Depression Scale (GDS-30). A cut-off of ≥10 was selected based on the widely reported optimal screening thresholds (9–11) identified in international meta-analyses [5,6]. According to the TYPA 2023 methodology, GDS-30 scores of 0–9 indicate no depression, scores of 10–19 indicate mild depression, and scores ≥20 indicate severe depression. The Turkish adaptation study provides evidence for reliability and construct validity but does not propose a cut-off value [7]. For the purposes of logistic regression analysis, participants with GDS-30 scores ≥10 (mild or severe depressive symptoms) were classified as having depressive symptoms, whereas those scoring <10 were classified as non-depressed. It should be noted that the GDS-30 is a screening instrument rather than a diagnostic tool; therefore, the outcome used in this study reflects depressive symptomatology and not clinically diagnosed depressive disorder.

Independent Variables: Independent variables were selected based on conceptual frameworks of late-life depression integrating sociodemographic vulnerability, health status, functional capacity, socioeconomic strain, healthcare access, and psychosocial experiences related to ageing. Sociodemographic factors included age group, sex, marital status (married vs. unmarried), and educational attainment (low, middle, high). Health-related determinants included self-rated health (good, moderate, poor), presence of chronic diseases, possession of an official disability report, and functional limitations across TYPA disability domains: vision, hearing, mobility, grasping, learning/cognitive, and speech difficulties (each coded as yes/no). These functional limitation variables were derived from questions based on the Washington Group framework on functioning and disability, which is consistent with the World Health Organization International Classification of Functioning, Disability and Health (ICF). Socioeconomic indicators included perceived change in household expenditures (increased, stable, decreased/irrelevant). Healthcare barriers were assessed using two TYPA 2023 items addressing (1) transportation to a health institution/organization or hospital and (2) communication with health professionals, including physicians, nurses, and administrative personnel. Both items used binary response categories (yes/no). Psychosocial ageing perceptions were measured using two TYPA 2023 items: (1) “Do you think your age prevents you from doing the things you want to do?” and (2) “Elderly people are discriminated in society.” Both items were coded as yes/no, with an additional ‘no idea’ option.

### Statistical analysis

All analyses accounted for the complex sampling design of TYPA 2023. Sampling weights (v181) and regional strata based on the 12 NUTS-1/IBBS regions were incorporated using the Complex Samples module of IBM SPSS Statistics 27.0. The sampling weights provided by TurkStat allow the survey respondents to represent the national population of older adults in Turkey. Therefore, although the analytical sample consisted of 10,348 participants, weighted estimates correspond to approximately 7.6 million community-dwelling adults aged 65 years and older at the national level. The survey weights provided by TurkStat incorporated design weights, non-response adjustments, calibration procedures, and population inflation factors. Descriptive statistics were reported as weighted frequencies and percentages for categorical variables and as weighted means for continuous variables.

Bivariate associations between depressive symptoms and categorical predictors were evaluated using the design-based Pearson chi-square test, which adjusts for sampling weights and stratification.

To identify independent determinants of depressive symptoms, Complex Samples Binary Logistic Regression was conducted using a hierarchical conceptual modeling strategy:

Model 1 (Core sociodemographic model): sex, age group, marital status, and educational level.

Model 2 (Health and functional limitations model): all variables in Model 1 plus self-rated health, chronic disease status, disability report, vision difficulty, hearing difficulty, walking difficulty, grasping difficulty, learning difficulty, and speech difficulty.

Model 3 (Final extended model): all variables in Model 2 plus difficulty reaching the hospital*,* difficulty communicating with healthcare workers*,* age-related restriction*, and* perceived exclusion of older adults, and expenditure change.

All categorical variables were dummy-coded. Survey-adjusted odds ratios (ORs) with 95% confidence intervals (CIs) were reported. Model fit and the contribution of each predictor were evaluated using design-based Wald F statistics and pseudo R^2^ coefficients (Cox–Snell, Nagelkerke, McFadden). A two-sided p-value <0.05 was considered statistically significant.

Multicollinearity among predictors was assessed using Variance Inflation Factors (VIFs). All VIF values were below 3.0 (range: 1.01–2.52), indicating no evidence of problematic multicollinearity among variables included in the fully adjusted model.

Model 3 was defined as the final fully adjusted model. All variables identified a priori from the conceptual framework were retained in the final model regardless of statistical significance to preserve theoretical consistency and minimize omitted-variable bias.

The original wording of all TYPA 2023 questionnaire items used in the present study is provided in S1 Appendix.

### Ethical considerations

This study was based on secondary analysis of anonymized microdata obtained through the Turkish Statistical Institute (TurkStat) Microdata Access Program (Application No. 596798). Access to the Turkey Older Persons Profile Survey (TYPA 2023) dataset was granted in accordance with the Turkish Statistical Law No. 5429 and the regulations governing the use of official statistical microdata.

The TYPA 2023 dataset contains no direct personal identifiers, and all analyses were conducted using fully anonymized data provided by TurkStat. Researchers had no access to information that could enable identification of individual participants.

As the present study involved secondary analysis of anonymized survey data, did not involve direct contact with participants, and did not include the collection of new personal data, additional ethics committee approval was not required for this secondary analysis of fully anonymized microdata under applicable national regulations. All analyses were conducted in accordance with the data confidentiality, security, and data-use requirements established by TurkStat, including restrictions on redistribution of the microdata to third parties.

## Results

Overall, the sample represented approximately 7.6 million older adults in Turkey. The weighted mean age of participants was 72.8 years (SE = 0.07). Individuals aged 65–74 years constituted 67.0% of the population, followed by those aged 75–84 years (27.0%) and ≥85 years (6.0%). Women represented 55.1% of older adults. Most were married (63.2%) or widowed (32.0%). Educational attainment was low: 30.6% had no formal education and 46.8% had completed primary school.

Overall health status was moderate to poor for the majority—50.4% rated their health as “moderate” and 26.1% as “poor/very poor.” Chronic disease was highly prevalent (78.3%). Functional limitations were frequent, including impairments in vision (53.9%), hearing (37.9%), walking (66.2%), grasping (68.2%), learning (47.4%), and speech (10.8%). A disability report was present in 7.4% of the population.

More than half reported increased household expenditures in the past three years (54.9%). Barriers to healthcare access were common: 30.8% had difficulty reaching a hospital and 16.3% experienced communication problems with healthcare workers. Psychosocial ageing-related concerns were also widespread, with 54.7% reporting age-related restrictions and 22.1% reporting perceived exclusion.

Based on weighted estimates, 46.2% screened positive for depressive symptoms, and the mean GDS-30 score was 10.20 (SE = 0.07) (Table 1).

**Table 1:** Weighted characteristics of older adults in the study sample (overall n = 10,348; valid n varies by item).

Variable (valid n)	Categories and distributions, n (%)
Age group	**65–74 years —** 67.0%, **75–84 years —** 27.0%, **≥85 years —** 6.0%
Sex (n = 10,348)	**Female —** 55.1%, **Male —** 44.9%
Marital status (n = 10,348)	**Married —** 63.2%, **Widowed —** 32.0%, **Divorced —** 3.4%, **Never married —** 1.3%
Education level (n = 10,348)	**No formal education —** 30.6%, **Primary school —** 46.8%, **Middle school —** 5.7%, **High school —** 8.2%, **Higher education —** 8.7%
Change in expenditures (3 years) (n = 10,348)	**Increased —** 54.9%, **Unchanged —** 26.4%, **Decreased —** 15.7%
Self-rated health (n = 10,348)	**Good/very good —** 23.5%, **Moderate —** 50.4%, P**oor/very poor —** 26.1%
Chronic disease (n = 10,348)	**Yes —** 78.3%, **No —** 21.7%
Disability report (n = 10,348)	**Yes —** 7.4%, **No —** 92.6%
Difficulties – Yes (n = 10,348)	**Vision difficulty —** 53.9%, **Hearing difficulty —** 37.9%, **Walking difficulty —** 66.2%, **Grasping difficulty —** 68.2%, **Learning difficulty —** 47.4%, **Speech difficulty —** 10.8%
Difficulty reaching hospital (n = 10,312)	**Yes** — 30.8%, **No** — 69.2%
Difficulty communicating with healthcare workers (n = 10,292)	**Yes** — 16.3%, **No** — 83.7%
Age-related restriction (n = 10,348)	**Yes** — 54.7%, **No** — 42.0%, **Not relevant** — 3.3%
Perceived exclusion of older adults (n = 10,348)	**Yes** — 22.1%, **No** — 69.0%, **No idea** — 8.9%
Depressive symptoms (n = 10,348)	**Yes** — 46.2%, **No** — 53.8%

In the design-weighted bivariate analyses, all sociodemographic, health-related, functional, and healthcare-access variables were significantly associated with depressive symptoms (all p < 0.001; see Supplementary Table 1 in S2 File for Rao–Scott χ^2^ tests).

Table 2 summarizes the results of the weighted hierarchical multivariable logistic regression models. In the multivariable survey-weighted logistic regression models, several sociodemographic, health-related, functional, and psychosocial factors remained independently associated with depressive symptoms. In the first model, older age showed a graded association with depressive symptoms, with adults aged 75–84 and those aged 85 years or older demonstrating significantly higher odds of depressive symptoms compared with those aged 65–74 years; however, these age effects were no longer significant after adjustment for health and functional indicators in Models 2 and 3. Female sex consistently remained a significant predictor across all models, with women exhibiting approximately 45–60% higher odds of depressive symptoms compared with men. Lower educational attainment also emerged as a strong determinant, and individuals with low education continued to show markedly elevated odds of depressive symptoms in the fully adjusted model. Being unmarried similarly maintained a significant association with depressive symptoms.

**Table 2:** Weighted hierarchical multivariable logistic regression models identifying factors associated with depressive symptoms.

Predictor	Model 1 OR (95% CI), p	Model 2 OR (95% CI), p	Model 3 OR (95% CI), p
Age group (ref = 65–74 years)			
75–84 years	1.419 (95% CI: 1.283–1.568), < 0.001	1.038 (95% CI: 0.929–1.160), 0.178	0.983 (95% CI: 0.877–1.103), 0.748
85 + years	2.204 (95% CI: 1.805–2.691), < 0.001	1.159 (95% CI: 0.935–1.436), 0.324	1.074 (95% CI: 0.857–1.345), 0.748
Sex (ref = Male)			
Female	1.641 (95% CI: 1.49–1.80), < 0.001	1.449 (95% CI: 1.303–1.611), < 0.001	1.438 (95% CI: 1.288–1.606), < 0.001
Educational level (ref = High)			
Low	2.926 (95% CI: 2.45–3.49), < 0.001	1.615 (95% CI: 1.334–1.956), < 0.001	1.551 (95% CI: 1.275–1.887), < 0.001
Middle	1.476 (95% CI: 1.20–1.82), < 0.001	1.205 (95% CI: 0.967–1.502), 0.096	1.204 (95% CI: 0.962–1.508), 0.096
Marital status (ref = Married)			
Unmarried	1.368 (95% CI: 1.24–1.51), < 0.001	1.262 (95% CI: 1.131–1.408), < 0.001	1.243 (95% CI: 1.108–1.393), < 0.001
Self-rated health (ref = Good)			
Poor	—	3.607 (95% CI: 3.065–4.246), < 0.001	3.191 (95% CI: 2.695–3.779), < 0.001
Moderate	—	1.504 (95% CI: 1.312–1.725), < 0.001	1.423 (95% CI: 1.235–1.640), < 0.001
Chronic disease (ref = No)	—	1.101 (95% CI: 0.963–1.258), 0.159	1.106 (95% CI: 0.962–1.271), 0.156
Disability report (ref = No)	—	1.639 (95% CI: 1.356–1.983), < 0.001	1.625 (95% CI: 1.334–1.980), < 0.001
Vision difficulty (ref = No)	—	1.421 (95% CI: 1.288–1.568), < 0.001	1.338 (95% CI: 1.207–1.482), < 0.001
Hearing difficulty (ref = No)	—	1.247 (95% CI: 1.121–1.387), < 0.001	1.179 (95% CI: 1.055–1.316), 0.004
Walking difficulty (ref = No)	—	1.086 (95% CI: 0.930–1.268), 0.296	1.004 (95% CI: 0.855–1.179), 0.965
Grasping difficulty (ref = No)	—	1.517 (95% CI: 1.294–1.778), < 0.001	1.388 (95% CI: 1.178–1.636), < 0.001
Learning difficulty (ref = No)	—	1.976 (95% CI: 1.780–2.193), < 0.001	1.834 (95% CI: 1.644–2.045), < 0.001
Speech difficulty (ref = No)	—	1.285 (95% CI: 1.096–1.508), 0.002	1.331 (95% CI: 1.126–1.573), 0.001
Perceived age-related restriction (ref = No)			
Yes	—	—	2.029 (95% CI: 1.827–2.253), < 0.001
Not relevant	—	—	1.814 (95% CI: 1.345–2.447), < 0.001
Perceived exclusion (ref = No)			
Yes	—	—	1.348 (95% CI: 1.196–1.519), < 0.001
No idea	—	—	1.036 (95% CI: 0.870–1.233), 0.563
Difficulty reaching hospital	—	—	1.386 (95% CI: 1.236–1.554), < 0.001
Communication problems with staff	—	—	1.554 (95% CI: 1.337–1.806), < 0.001
Expenditure change (ref = Decreased)	—	—	
Unchanged	—	—	0.680 (95% CI: 0.582–0.793), < 0.001
Increased	—	—	0.671 (95% CI: 0.583–0.772), < 0.001

Note. All predictors specified in the theoretical model were retained in the final model regardless of statistical significance.

Health-related indicators demonstrated the largest effect sizes. Poor self-rated health was associated with more than a threefold increase in the odds of depressive symptoms, and moderate health also remained significant. The presence of an official disability report was independently associated with higher depressive symptoms. Several functional limitations—including difficulties with vision, hearing, grasping, learning and speech—retained strong and statistically significant associations in the final model, even after adjustment for sociodemographic and health factors. In contrast, chronic disease status and walking difficulty were not independently associated with depressive symptoms in the fully adjusted model.

Psychosocial ageing perceptions showed some of the strongest associations in the fully adjusted model. Importantly, these associations remained significant even after adjusting for extensive health and functional limitations, indicating that psychosocial and healthcare-related factors contribute independently to depressive symptoms in later life. Older adults who reported age-related restrictions had nearly twice the odds of depressive symptoms, and similar elevated odds were observed among those who expressed uncertainty regarding such restrictions. Perceived exclusion was independently associated with higher odds of depressive symptoms among respondents reporting exclusion. Barriers to healthcare access contributed additionally to depressive risk; difficulty reaching a hospital and communication problems with healthcare staff were both linked to substantially higher odds of depressive symptoms after full adjustment.

All three survey-weighted logistic regression models demonstrated acceptable overall performance. Design-based Wald F tests indicated that each model was statistically significant (all p < 0.001), and the explanatory power increased progressively across models. Nagelkerke R^2^ rose from 11% in Model 1–29% in Model 2 and reached 32% in the final model (Model 3). The largest increase in explanatory power occurred after adding health and functional indicators (Model 2), while psychosocial perceptions of ageing and healthcare access barriers contributed additional explanatory value in the final model.

Sampling weights and 12 regional strata were incorporated in all analyses, ensuring valid design-adjusted standard errors. No convergence issues or separation problems were detected, indicating stable and reliable parameter estimates (Supplementary Table 2 in S2 File).

## Discussion

Using nationally representative data from the Turkey Older Persons Profile Survey (TYPA 2023), this study examined correlates of depressive symptoms among adults aged ≥65 years. Nearly half of the sample screened positive for depressive symptoms using the GDS-30 (cut-off ≥10). Depressive symptoms were associated with female sex, lower educational attainment, unmarried status, poor self-rated health, multiple domains of functional and sensory difficulty, economic strain, barriers in using health services, and negative psychosocial perceptions of ageing. Model performance improved substantially after incorporating health, disability and psychosocial variables, indicating that depressive symptoms in later life co-occur with a multidimensional set of structural, health-related and contextual factors. Notably, psychosocial perceptions of ageing and healthcare access barriers remained significant even after adjusting for extensive health and functional limitations.

It is important to note that the outcome in the present study was defined using the GDS-30, a screening instrument designed to identify depressive symptomatology rather than clinically diagnosed depressive disorder. Therefore, the observed prevalence reflects the proportion of older adults screening positive for depressive symptoms (GDS-30 ≥ 10) and should not be interpreted as the prevalence of clinically diagnosed depression.

### Comparison with previous research

The observed prevalence falls within the broad range reported in prior studies using the Geriatric Depression Scale in Turkey and internationally [9,18]. Variation in cut-off values and study settings likely contributes to this spread. The prevalence observed in our study is consistent with findings from a recent meta-analysis including 57,486 older adults, which reported a pooled prevalence of depression of 31.7% worldwide. Furthermore, studies using the Geriatric Depression Scale reported even higher prevalence estimates, ranging from 35.7% (GDS-15) to 40.6% (GDS-30) [15]. Therefore, although the prevalence observed in the present nationally representative sample is relatively high, it is comparable to estimates reported in studies using similar screening instruments and suggests that depressive symptoms constitute a substantial public health concern among older adults in Turkey. Findings should be interpreted as reflecting screening-positive depressive symptoms rather than clinical diagnoses [19].

### Sociodemographic correlates

Female sex, lower education and being unmarried remained independently associated with depressive symptoms, consistent with a large body of literature across diverse contexts [15,18]. The higher odds observed among women are also consistent with international evidence indicating that depressive symptoms are approximately 1.0–1.7 times more common among older women than older men across a variety of settings [9]. The lack of an age gradient after adjusting for health and functional indicators suggests that the observed raw association between age and depressive symptoms may reflect declining intrinsic capacity rather than chronological age per se [20]. Selective survival and selective participation among the oldest-old may also contribute to attenuated age differences [9,21].

### Health, functional and sensory correlates

Poor self-rated health showed the strongest association with depressive symptoms. This aligns with evidence identifying self-rated health as one of the most robust indicators of depressive symptomatology and future symptom development [1]. Multiple functional and sensory limitations—including difficulties with vision, hearing, grasping, learning and speech—were independently associated with depressive symptoms. These findings parallel research linking declines in physical and cognitive functioning with heightened depressive symptom levels [22]. Using nationally representative data from India, Ansari et al. reported that limitations in activities of daily living (ADL) and instrumental activities of daily living (IADL) accounted for approximately 21–23% of the association between multimorbidity and depressive symptoms among older adults, highlighting the importance of functional impairment as a pathway to depression [23].

Chronic disease did not remain significantly associated with depressive symptoms after adjusting for health perceptions and functional indicators. This pattern is consistent with studies showing that the subjective and functional consequences of multimorbidity, rather than diagnostic counts alone, account for much of its association with depressive symptoms [23,24].

### Economic and psychosocial correlates

Indicators of economic strain, including decreased household expenditures, remained independently associated with depressive symptoms. Prior studies in Turkey and elsewhere similarly demonstrate links between financial hardship, unmet needs and late-life depressive symptoms [1,18].

Psychosocial perceptions related to ageing—feeling restricted because of age and perceiving societal exclusion—showed some of the strongest associations in the final model. These findings are consistent with literature identifying perceived ageism and negative self-perceptions of ageing as important correlates of emotional well-being [23,25–27].

These findings highlight the importance of subjective ageing experiences and perceived social position in shaping mental health outcomes in later life.

### Healthcare-related correlates

Difficulty accessing healthcare facilities and communicating with healthcare workers was associated with higher odds of depressive symptoms. Barriers in health service use have similarly been linked to poorer psychological well-being in older adults [1]. Given the high burden of chronic disease in this population, such barriers may be associated with unmet psychosocial needs [12]. They may also be associated with reduced opportunities for early detection and management of depressive symptoms.

### Interpretation of disability report findings

The association between having an official disability report and depressive symptoms may reflect more severe or persistent impairment not fully captured by functional measures. However, disability certificates can also facilitate access to benefits and services, potentially reducing psychological distress [28]. The present cross-sectional design does not allow these pathways to be disentangled.

### Strengths and limitations

Strengths include the use of a large, nationally representative sample, a validated screening tool (GDS-30), and a hierarchical modelling strategy incorporating diverse domains.

Limitations include the cross-sectional design, which precludes causal inference; the self-reported nature of all measures; and the absence of certain relevant factors such as detailed social support, psychiatric history, antidepressant use and neighbourhood characteristics. The unavailability of these variables may have resulted in residual confounding and should be considered when interpreting the observed associations. Measures of economic strain and healthcare access may not fully capture these multidimensional constructs. Although model performance improved after adding health, disability and psychosocial variables (Nagelkerke R^2^ up to ~0.32), much of the variance remains unexplained, suggesting the presence of additional unmeasured factors influencing depressive symptom development in later life. Additionally, proxy respondents were excluded because the GDS-30 was administered only to participants who completed the interview themselves. Consequently, older adults with severe cognitive, physical, or communication impairments may be underrepresented in the analytical sample, which could have influenced both prevalence estimates and observed associations.

## Conclusions

This nationally representative study demonstrates that screening-positive depressive symptoms are highly prevalent among older adults in Turkey and are associated with a wide range of sociodemographic, health, functional, economic, psychosocial and healthcare-related factors. These patterns suggest that late-life depressive symptoms reflect intersecting structural, functional and psychosocial vulnerabilities rather than biomedical factors alone.

Supporting functional independence, reducing economic hardship, improving age-friendly and accessible healthcare, and addressing ageism may help alleviate the burden of depressive symptoms among older adults. Longitudinal studies are needed to clarify temporal relationships and inform targeted prevention and intervention strategies.

## Supporting information

S1 Appendix Description of the Türkiye Older Persons Profile Survey (TYPA 2023) and Operational Definitions of Variables Used in the Present Study. (DOCX)

S2 File Supplementary Tables. Contains Supplementary Table 1 (Design-based bivariate associations between predictors and depressive symptoms using Rao–Scott adjusted chi-square tests) and Supplementary Table 2 (Model performance indicators for the three survey-weighted logistic regression models).
(DOCX)
