# Supplementary material for: Depressive symptoms among older adults in Turkey: Evidence from a nationally representative ageing survey
Source: PLoS One. 2026 Jul 30;21(7):e0354722. doi: 10.1371/journal.pone.0354722 (PMC13422882; doi:10.1371/journal.pone.0354722)
Supplement: S1 Appendix — (DOCX) [file pone.0354722.s001.docx]

S1 Appendix. Description of the Türkiye Older Persons Profile Survey (TYPA 2023) and Operational Definitions of Variables Used in the Present Study

Introduction

The present study utilized data from the Türkiye Older Persons Profile Survey (TYPA 2023), a nationally representative cross-sectional survey conducted by the Turkish Statistical Institute (TurkStat) in collaboration with the Ministry of Family and Social Services. Fieldwork was carried out between 23 October and 18 December 2023 throughout Türkiye. The survey collected information on demographic characteristics, socioeconomic conditions, health, functional status, social participation, discrimination, and well-being among older adults.

This appendix provides the original wording of questionnaire items used to derive variables included in the present analyses and describes the operational definitions applied during data processing.

1. Outcome Variable

1.1 Depressive Symptoms (Geriatric Depression Scale-30)

Depressive symptoms were assessed using the 30-item Geriatric Depression Scale (GDS-30), administered as part of TYPA 2023.

According to the TYPA methodology:

• 0–9 points = Not depressed
• 10–19 points = Mild depression
• ≥20 points = Severe depression

For the present analyses, participants scoring ≥10 points were classified as having depressive symptoms.

1. Sociodemographic Variables

Age, sex, marital status, and educational level were obtained directly from the TYPA 2023 questionnaire and categorized as described in the Methods section.

3. Health-Related Variables

3.1 Self-Rated Health

Original survey item:
"How would you describe your general health status?"

Operational definition:
Responses were categorized as good, moderate, and poor.

3.2 Chronic Disease

Original survey item:
"Do you have chronic diseases such as hypertension, diabetes, heart disease, cancer, kidney failure, stroke, hepatitis, asthma, etc.?"

Operational definition:
Yes / No

1. Functional Limitations

The following questions were derived from the Washington Group framework on functioning and disability.

4.1 Vision Difficulty

Original survey item:

"Do you have difficulty seeing, even if wearing glasses or contact lenses?"

Response options:
• No difficulty
• Some difficulty
• A lot of difficulty
• Cannot see at all

Operational definition:
Responses were dichotomized as no difficulty versus any difficulty.

4.2 Hearing Difficulty

Original survey item:

"Do you have difficulty hearing, even if you use a hearing aid or implant?"

Response options:
• No difficulty
• Some difficulty
• A lot of difficulty
• Cannot hear at all

Operational definition:
Responses were dichotomized as no difficulty versus any difficulty.

4.3 Speech Difficulty

Original survey item:

"Do you have difficulty speaking (such as speech impairment, lallopathy, stammering, etc.)?"

Response options:
• No difficulty
• Some difficulty
• A lot of difficulty
• Cannot speak at all

Operational definition:
Responses were dichotomized as no difficulty versus any difficulty.

4.4 Walking Difficulty

Original survey item:

"Do you have difficulty walking or climbing steps?"

Response options:
• No difficulty
• Some difficulty
• A lot of difficulty
• Cannot do it at all

Operational definition:
Responses were dichotomized as no difficulty versus any difficulty.

4.5 Grasping Difficulty

Original survey item:

"Do you have difficulty holding or lifting something?"

Response options:
• No difficulty
• Some difficulty
• A lot of difficulty
• Cannot do it at all

Operational definition:
Responses were dichotomized as no difficulty versus any difficulty.

4.6 Learning Difficulty

Original survey item:

"Do you have difficulty learning, doing simple calculations, remembering, or concentrating when compared to your peers?"

Response options:
• No difficulty
• Some difficulty
• A lot of difficulty
• Cannot do it at all

Operational definition:
Responses were dichotomized as no difficulty versus any difficulty.

1. Healthcare Access Variables

5.1 Difficulty Reaching Healthcare Services

Original survey item:
"Transportation to a health institution/organization or hospital"

5.2 Communication Problems with Healthcare Professionals

Original survey item:
"Communication with health professionals; doctors, nurses, secretaries, etc."

1. Social and Ageing-Related Variables

6.1 Perceived Age-Related Restriction

Original survey item:
"Do you think your age prevents you from doing the things you want to do?"

Response options:
• Yes
• No
• Not relevant

6.2 Perceived Exclusion of Older Adults

Original survey item:
"Elderly people are discriminated in the society."

Response options:
• Yes
• No
• No idea

1. Economic Variable

7.1 Change in Expenditures During the Last Three Years

Original survey item:
"What is the status of your expenses in the last three years?"

Response options:
• Increased
• Stayed the same
• Decreased
