# Supplementary material for: Depressive symptoms among older adults in Turkey: Evidence from a nationally representative ageing survey
Source: PLoS One. 2026 Jul 30;21(7):e0354722. doi: 10.1371/journal.pone.0354722 (PMC13422882; doi:10.1371/journal.pone.0354722)
Supplement: S2 File — Contains Supplementary Table 1 (Design-based bivariate associations between predictors and depressive symptoms using Rao–Scott adjusted chi-square tests) and Supplementary Table 2 (Model performance indicators for the three survey-weighted logistic regression models). (DOCX) [file pone.0354722.s002.docx]

| Supplementary Table 1**.** Design-based bivariate associations between predictors and depressive symptoms using Rao–Scott adjusted chi-square tests **(n = 10,348)** | | |
| --- | --- | --- |
| **Variable** | **Rao–Scott χ² (df); Adjusted F (df1, df2)** | **p-value** |
| Sex | 357.355 (1); F = 307.783 (1, 10336) | <.001 |
| Age group | 232.939 (2); F = 100.606 (2, 20671.77) | <.001 |
| Marital status | 316.822 (2.995); F = 84.280 (2.995, 30956.56) | <.001 |
| Education level | 774.995 (7.897); F = 87.151 (7.897, 81627.89) | <.001 |
| Change in expenditures | 124.324 (3); F = 36.384 (3, 31003.30) | <.001 |
| Self-rated health | 1337.470 (3.985); F = 290.948 (3.985, 41192.35) | <.001 |
| Chronic disease | 330.075 (1); F = 285.458 (1, 10336) | <.001 |
| Vision difficulty | 607.191 (2.998); F = 178.339 (2.998, 30989.60) | <.001 |
| Hearing difficulty | 473.133 (2.988); F = 129.711 (2.988, 30882.98) | <.001 |
| Walking difficulty | 1381.368 (2.993); F = 410.047 (2.993, 30938.92) | <.001 |
| Speech difficulty | 270.682 (2.990); F = 79.895 (2.990, 30904.41) | <.001 |
| Grasping difficulty | 1431.989 (2.999); F = 410.864 (2.999, 30995.43) | <.001 |
| Learning/remembering difficulty | 1329.667 (2.997); F = 380.372 (2.997, 30977.79) | <.001 |
| Disability report | 183.247 (1); F = 156.981 (1, 10336) | <.001 |
| Difficulty reaching a hospital | 529.845 (1.982); F = 248.546 (1.982, 20489.26) | <.001 |
| Communication problems with staff | 364.129 (1.979); F = 167.019 (1.979, 20453.81) | <.001 |
| Age-related restriction | 782.978 (2); F = 345.590 (2, 20667.07) | <.001 |
| Perceived exclusion | 99.853 (1.997); F = 43.592 (1.997, 20643.91) | <.001 |
| ***Note.*** *All p-values are based on the adjusted F statistic (second-order Rao–Scott correction). Weighted estimates were used in all analyses.* | | |

| Supplementary Table 2. Model performance indicators for the three survey-weighted logistic regression models | | | |
| --- | --- | --- | --- |
| **Model** | **Design-based Wald F (df)** | **p-value** | **Nagelkerke R²** |
| Model 1 | | | |
| Sociodemographic | 109.97 (df = 10,331) | <.001 | 0.11 |
| Model 2 | | | |
| (Health + Functional) | 104.14 (df = 10,321) | <.001 | 0.29 |
| Model 3 | | | |
| Final Extended Model | 73.33 (df = 10,002) | <.001 | 0.32 |
| ***Notes:*** *All models incorporate sampling weights and 12 IBBS/NUTS-1 regional strata. Wald F values represent the overall significance of each model after accounting for the complex survey design. Nagelkerke R² shows the progressive increase in explained variance across hierarchical models.* | | | |
